# Phase I Evaluation of Intravenous Ascorbic Acid in Combination with Gemcitabine and Erlotinib in Patients with Metastatic Pancreatic Cancer

**DOI:** 10.1371/journal.pone.0029794

**Published:** 2012-01-17

**Authors:** Daniel A. Monti, Edith Mitchell, Anthony J. Bazzan, Susan Littman, George Zabrecky, Charles J. Yeo, Madhaven V. Pillai, Andrew B. Newberg, Sandeep Deshmukh, Mark Levine

**Affiliations:** 1 Myrna Brind Center of Integrative Medicine, Thomas Jefferson University, Philadelphia, Pennsylvania, United States of America; 2 Department of Medical Oncology, Thomas Jefferson University, Philadelphia, Pennsylvania, United States of America; 3 Department of Surgery and the Jefferson Pancreas, Biliary, and Related Cancer Center, Thomas Jefferson University, Philadelphia, Pennsylvania, United States of America; 4 Department of Radiology, Thomas Jefferson University, Philadelphia, Pennsylvania, United States of America; 5 Molecular and Clinical Nutrition Section, National Institute of Diabetes and Digestive and Kidney Diseases, National Institutes of Health, Bethesda, Maryland, United States of America; University Clinic of Navarra, Spain

## Abstract

**Background:**

Preclinical data support further investigation of ascorbic acid in pancreatic cancer. There are currently insufficient safety data in human subjects, particularly when ascorbic acid is combined with chemotherapy.

**Methods and Findings:**

14 subjects with metastatic stage IV pancreatic cancer were recruited to receive an eight week cycle of intravenous ascorbic acid (three infusions per week), using a dose escalation design, along with standard treatment of gemcitabine and erlotinib. Of 14 recruited subjects enrolled, nine completed the study (three in each dosage tier). There were fifteen non-serious adverse events and eight serious adverse events, all likely related to progression of disease or treatment with gemcitabine or erlotinib. Applying RECIST 1.0 criteria, seven of the nine subjects had stable disease while the other two had progressive disease.

**Conclusions:**

These initial safety data do not reveal increased toxicity with the addition of ascorbic acid to gemcitabine and erlotinib in pancreatic cancer patients. This, combined with the observed response to treatment, suggests the need for a phase II study of longer duration.

**Trial Registration:**

Clinicaltrials.gov NCT00954525

## Introduction

Pancreatic cancer survival continues to be amongst the shortest of all cancers, and new therapies are urgently needed. The majority of patients with pancreatic cancer already have metastatic disease at clinical presentation [1], and many patients progress quickly even with standard treatment of gemcitabine alone or gemcitabine plus erlotinib [2], [3], [4].

Ascorbic acid (ascorbate, vitamin C) in cancer care has had a labyrinthine history [5]. Several decades ago, observational and anecdotal clinical data obtained by Cameron and Pauling suggested an unexpected increase in survival in some patients who received 10 grams of ascorbic acid daily compared to retrospective controls [6], [7]. However, two double-blinded placebo-controlled trials showed no efficacy of the same ascorbic acid dose [8], [9], and thus, ascorbate was dismissed from therapeutic consideration in 1985 [10]. A more recent review and analysis of patients receiving oral doses of ascorbic acid demonstrated no benefit in cancer patients [11].

Since then, renewed interest in ascorbic acid and cancer treatment arose serendipitously from clinical pharmacokinetics studies of ascorbic acid in healthy adults [12], [13]. In those studies, to determine true bioavailability, subjects received both oral and intravenous ascorbate. When ascorbic acid was given intravenously in doses above 0.5 grams, it was found that the usual tight control of ascorbic acid concentrations with oral doses was bypassed. Only intravenous administration resulted in very high ascorbic acid concentrations until renal excretion restored homeostasis. With these pharmacokinetics data as background, investigators revisited the earlier work on cancer and found that in the studies by Cameron and Pauling, patients received both intravenous as well as oral ascorbate, while patients from the later studies received only oral doses.

Detailed pharmacokinetics studies in humans and animals have confirmed that intravenous ascorbate in pharmacologic doses can produce peak plasma concentrations that are several hundred fold higher than those possible from maximal oral doses [12], [13]. In cell and animal experiments, such pharmacologic concentrations of ascorbate kill a number of cancer cell types, but not normal cells, and decrease tumor growth in mice [14], [15]. In humans, plasma ascorbate concentrations produced by intake of vitamin C rich foods (fruits and vegetables) are usually <0.1 mM, and by higher intake from supplements are <0.15 mM [13], [16], [17]. In rodents, baseline plasma ascorbate concentrations are approximately 0.05 mM. When parenteral pharmacologic ascorbate doses are administered to animals or humans, peak plasma concentrations are as high as 30 mM [15], [18]. Across this broad range of concentrations, ascorbate in plasma readily diffuses into extracellular fluid [15], [19]. At extracellular fluid ascorbate concentrations above 3–4 mM, hydrogen peroxide concentrations above 5 µM are detectable in this fluid but not in blood [14], [15], [19]. Such hydrogen peroxide concentrations do not otherwise occur with physiologic ascorbate concentrations. Hydrogen peroxide plus ascorbate in extracellular fluid results in formation of reactive oxygen species, which are selectively toxic to cancer cells but not normal tissues [14], [15]. Thus, pharmacologic ascorbate is a pro-drug for production of sustained concentrations of hydrogen peroxide in extracellular fluid but not blood [14], [15], [19].

There are limited human data on the use of pharmacologic ascorbate, despite surprisingly current wide use by practitioners of complementary and alternative medicine [5]. One clinical safety study of pharmacologic ascorbate in patients with a variety of advanced cancers did not reveal untoward effects [18].

Pancreatic cancer is sensitive to pharmacologic ascorbate both in vitro and in animal models [15]. Emerging evidence in both model systems indicates that ascorbate has synergistic effects with gemcitabine [20]. When pharmacologic ascorbate was combined with gemcitabine, synergy was observed in all eight cell lines tested in vitro. In mouse models, ascorbate- gemcitabine combinations were more effective at inhibiting tumor growth compared to gemcitabine alone and also produced gemictabine dose-sparing effects.

Given what is known about the relative safety of pharmacologic ascorbate and its potential for efficacy, coupled to the pressing need for new treatments, we conducted a phase I trial of intravenous ascorbate added to gemcitabine and erlotinib in patients with stage IV metastatic pancreatic ductal adenocarcinoma with the specific primary aim of assessing safety and the secondary aim of assessing response to treatment. We investigated adverse events, measured peak plasma ascorbic acid concentrations after infusions, and conducted imaging pre- and post-treatment.

## Methods

### Ethics Statements

This research was approved by the Thomas Jefferson University Institutional Review Board. Written informed consent, approved by the Thomas Jefferson University Institutional Review Board, was received from all patients who participated in the study. Clinical Investigation was conducted according to the principles expressed in the Declaration of Helsinki.

### Study design

**Figure 1:**
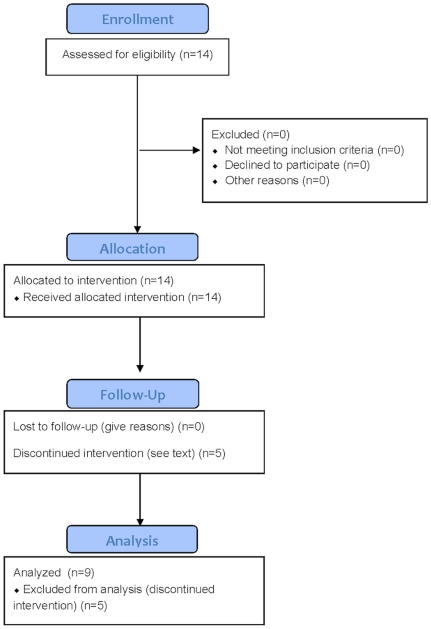
Study CONSORT flow diagram.

This was a phase I study of patients with histologically or cytologically confirmed metastatic stage IV pancreatic ductal adenocarcinoma, conducted at the Thomas Jefferson University and Hospital between July, 2009 and July, 2011. The study was an open-label, dose-escalating trial that utilized a 3+3+3 design (see Figure 1 for CONSORT diagram [21]). The protocol for the study and supporting CONSORT checklist are available as supporting information; see Protocol S1 and Checklist S1. Patients were referred by the study oncologists who were aware of the study inclusion/exclusion criteria. All referred patients are included in the data presented. However, it is possible that some patients who met study criteria were referred to another competing trial. Recruitment occurred July 2009 to July 2011.The first cohort received 50 grams intravenous ascorbate per infusion, the second cohort received 75 g/infusion, and the third cohort received 100 g/infusion. A cycle consisted of three infusions per week performed on separate days, for 8 weeks (maximum twenty-four infusions total per dose level). One hundred grams per infusion was the target ceiling dose for this study, because this dosage was estimated to produce a blood level high enough to achieve the proposed mechanism of action of elaborating hydrogen peroxide [22]. Fifty grams was chosen as the starting dosage based upon available safety data [5], [20]. Ascorbate blood levels were drawn immediately after the first target dose was reached and again at the final dose of the cycle for the patients in the 75 g and 100 g dosage tiers.

The following inclusion and exclusion criteria were used for subject recruitment into the study. Patients had newly diagnosed, stage IV pancreatic ductal adenocarcinoma, were not eligible for surgical resection, and had not yet been treated. The diagnosis was established by histology or cytology. Patients had an Eastern Cooperative Oncology Group (ECOG) Performance Status of 0–2. Laboratory requirements were an absolute neutrophil count ≥1,500/mm3, hemoglobin >8 g/dL, platelet ≥100,000/mm3, total bilirubin ≤1.5 mg/dL, creatinine ≤2.0 mg/dL, transaminases ≤2.5× upper limit, urine uric acid <1,000 mg/d, urine pH<6, and urine oxalate <60 mg/d. Patients were excluded if they had documented glucose-6-phosphate-dehydrogenase (G6PD) deficiency or a history of oxalate renal calculi, since high levels of ascorbic acid are known to cause hemolysis in G6PD deficient individuals and kidney stones in those patients with a preexisting risk for oxalate stones. Patients were excluded if they were currently receiving chemotherapy or radiation therapy or enrolled in other trials currently or in the preceding 1 month. Patients with co-morbid condition that would affect survival such as end stage congestive heart failure, unstable angina, myocardial infarction within 6 weeks of study, or chronic active hepatitis or cirrhosis also were excluded.

All subjects meeting eligibility criteria were enrolled to receive gemcitabine, erlotinib, and intravenous ascorbate as first line treatment. Gemcitabine was administered intravenously at a dose of 1000 mg/m^2^ over 30 minutes, on day 1, once weekly for 7 weeks followed by a 1 week rest. Erlotinib was given orally in a single daily dose of 100 mg per day for eight weeks.

Data were obtained regarding laboratory values and adverse events throughout the treatment period. Ascorbic acid concentrations in the serum were measured by HPLC with coulometric electrochemical detection [13]. Monitoring and adverse events were evaluated by standard National Cancer Institute (NCI) clinical criteria 3.0 [23]. At the beginning and end of the ascorbate treatment cycle, subjects were evaluated by x-ray computed tomography (CT) imaging of the chest, abdomen, and pelvis, for possible response to treatment based upon Response Evaluation Criteria In Solid Tumors (RECIST 1.0) criteria [24]. The expert radiologist who evaluated CT images was blinded to patient treatment.

### Ascorbic Acid Preparation

Ascorbic Acid Injection USP was supplied by Bioniche Pharma (Rosemont IL) as sterile solution of ascorbic acid in water for parenteral use. The product was supplied in sterile 50 mL single use glass ampoules. Each mL contained ascorbic acid 500 mg (2.84 mmol), edetate disodium 0.025%, and water for injection with pH (range 5.5 to 7.0) adjusted with sodium bicarbonate, hence providing ∼2.84 mmol sodium and an osmolality of 2.84+2.84 = 5.7 mOsm/mL.

## Results

### Patient Cohort

To meet the goal of 9 patients completing the trial, 14 patients were recruited from July, 2009 to July, 2011: 4 males and 10 females, mean age of 64.4±10.0 (range 47–81)(Table 1). All patients had stage IV pancreatic ductal adenocarcinoma. Nine patients (three in each dosage tier) received the full cycle of ascorbic acid plus gemcitabine and erlotinib, with full cycle defined as at least 24±6 ascorbic acid treatments for 8±1 weeks. These nine subjects were evaluated by CT imaging pre and post therapy. Five of the fourteen enrolled patients did not complete the study and therefore were not evaluable by CT imaging. Of these, two subjects (003 and 007) chose not to continue because it was too difficult to come in for the treatments, and three subjects died from rapid disease progression: subject 009 after 5 weeks of treatment; subject 011 after 3 weeks of treatment, and subject 013 after 1 week of treatment.

**Table 1:** Patient demographics and disease status.

ID	Age/Gender	Time Dx to Rx	ECOG Status	Dx (in addition to pancreatic primary mass)*	Dose Level (g/infusion)	Doses	Weeks	Weight Pre Rx(lbs)	Weight Post Rx**(lbs)
001	60 M	3 wks	0	Liver/lung/media	50	24	8	143	134
002	75 F	5 wks	2	Liver/retroper	50	24	8	143	134
003	81 F	5 wks	1	Locally adv/liver	50	3	1	100	----
004	64 F	4 wks	1	Liver	50	18	7	157	148
005	69 M	6 mon	1	Abdomen	75	23	8	167	157
006	66 F	2 wks	0	Media/retroper	75	22	8	224	202
007	47 F	6 wks	0	Liver/peritoneal	75	3	1	100	----
008	75 F	3 wks	1	Liver	75	21	7	156	144
009	51 M	3 mon	1	Liver/peritoneal	75	14	5*	162	168
010	48 F	3 wks	1	Liver	100	21	8	186	169
011	67 F	4 wks	1	Liver/peritoneal	100	9	3*	140	144
012	67 F	4 mon	1	Locally adv/bone	100	24	8	147	133
013	65 M	4 mon	2	Liver/peritoneal	100	3	1*	137	----
014	66 F	3 wks	1	Liver	100	24	8	148	141

Bone = bone metastases.

Liver = liver metastases.

Locally adv = locally advanced spread of cancer.

Abdomen = Metastases within the abdomen distant from the pancreas.

Lung = lung metastases.

Media = mediastinal metastases.

Peritoneal = peritoneal metastases.

Retroper = retroperitoneal nodes or metastases.

*Patient died during study.

**Weights were obtained on the first and last day of the ascorbic acid infusions.

### Safety and Adverse Events

When patients received intravenous ascorbic acid, they frequently reported mild lightheadedness or nausea which was expected from the osmotic load and resolved with eating and drinking. Overall, for the total cohort of 14 patients, there were 23 total adverse events with 8 being serious adverse events. The adverse events are shown in Table 2. All of these adverse events were most likely attributable to progression of disease or concomitant treatment with gemcitabine and/or erlotinib. Regarding the serious adverse events: one male subject was hospitalized with low hemoglobin due to an internal bleed and then was subsequently placed on hospice care. Two subjects were found to have a pulmonary embolism most likely related to the underlying pancreatic cancer that has a reported rate of pulmonary embolism between 20–50% [25]. Three subjects died from progression of the underlying cancer, as determined clinically and with confirmation by the data safety and monitoring board. One patient was hospitalized twice, once for anemia symptoms and once for a urinary tract infection which both resolved. A male subject was hospitalized with abdominal pain and ileus which in retrospect were present at study onset, and he received total parenteral nutrition and nasogastric tube feeds, but was finally put on hospice care before dying of the underlying cancer. None of these subjects received the full treatment with intravenous ascorbic acid. None of these adverse events appeared to be specifically related to the ascorbic acid treatment since each of these events is frequently observed in the normal progression of pancreatic cancer patients and/or gemcitabine and erlotinib treatment.

**Table 2:** Adverse Event Chart for all 14 patients (based on standard NCI criteria).

Adverse Event	Number of Events
**Low Platelets**	
Grade 1	6
Grade 2	2
**Low Hemoglobin**	
Grade 2	1
Grade 3	2
**Low Absolute Neutrophil Count**	
Grade 3	1
**Elevated Glucose**	
Grade 2	1
**Gastrointestinal**	
Ileus (Grade 3)	1
Discomfort (Grade 2)	1
Ascites (Grade 2)	1
**Infection**	
Conjunctival (Grade 2)	1
Urinary Tract Infection (Grade 3)	1
**Pulmonary Emboli**	
Grade 4	2
**Death**	
Grade 5	3

### Pharmacology

**Figure 2:**
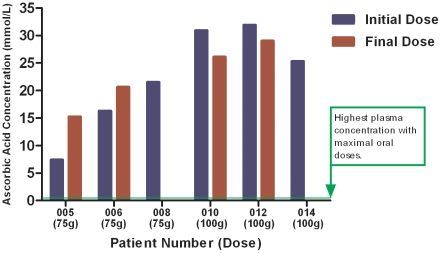
Peak plasma ascorbic acid concentrations in millimoles/L after the initial dose and final dose (measurements after the final dose were unavailable for patients 008 and 014). The green line represents the highest plasma concentration expected with maximally tolerated oral doses of ascorbic acid [13].

Ascorbic acid concentrations were measured immediately after infusion end in the six patients receiving the two upper dosage tiers of either 75 g or 100 g per infusion (see Figure 2). Millimolar concentrations were achieved as expected, particularly among those receiving 100 g per infusion. For these patients the plasma ascorbate level was between 25.3 and 31.9 millimoles/L. They had no increase in adverse events compared to the other dosage tiers or to what would be expected from gemcitabine and erlotinib alone or from progression of disease.

### Response to Treatment

**Figure 3:**
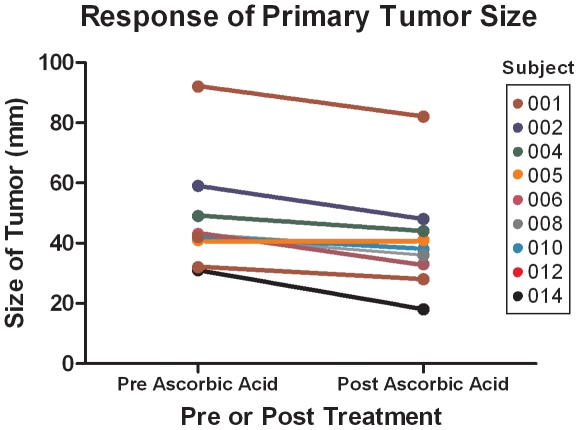
Tumor size initially and after 8 weeks of treatment with ascorbic acid, gemcitabine, and erlotinib for each of the patients who completed the study.

To assess treatment response by imaging, all nine patients who completed the protocol underwent pre- and post-treatment CT scans or PET-CT scans. Scans were evaluated, by an expert radiologist blinded to the clinical conditions of the patients, for the change in size of the primary tumor (Figure 3) as well as by RECIST 1.0 criteria (Table 3). As shown in Figure 3, eight of nine patients had a reduction in size of the primary tumor (with one patient having no change in size). By RECIST 1.0 criteria, 7 patients had stable disease and 2 patients had progressive disease (non-responders). In addition, 3 patients did not have post imaging results because they died before the end of the treatment period. With these patients included, the total for progressive disease (non-responders) is 5 patients. There were no distinguishing characteristics regarding the patients who died during the study compared to those who completed the study. Finally, although our analyses were focused on the eight week treatment period, we obtained additional data for preliminary survival assessments. The estimated mean progression free survival measured from the first day of treatment until evidence of progression was 89 days (SD 77 days) and the overall survival was 182 days (SD 155 days).

**Table 3:** Response to ascorbic acid plus gemcitabine based on CT findings in patients with scans pre and post the 8 week ascorbic acid treatment cycle plus gemcitabine/erlotinib (these data only include patients that completed both pre and post-treatment CT imaging and not the additional five patients who terminated participation early).

Patient (Dose)	Pancreatic Mass	Pancreatic Mass	% Change	Non-Target	RECIST Criteria
	Pre (mm)	Post (mm)	Primary Mass	Lesions	Response
001 (50 g)	31×18	18×16	−42%	Stable	SD
002 (50 g)	32×20	28×22	−13%	Progressed	PD
004 (50 g)	43×39	33×39	−23%	Stable	SD
005 (75 g)	43×19	38×21	−12%	Stable	SD
006 (75 g)	92×42	82×36	−11%	Stable	SD
008 (75 g)	41×38	41×38	0%	Progressed	PD
010 (100 g)	59×38	48×37	−19%	Stable	SD
012 (100 g)	49×49	44×42	−10%	Improved	SD
014 (100 g)	42×22	36×17	−14%	Improved	SD

SD = Stable Disease.

PD = Progressive Disease.

## Discussion

The goals of this phase I trial were to provide an initial safety evaluation of ascorbic acid added to gemcitabine and erlotinib in patients with stage IV pancreatic cancer, to measure whether predicted ascorbic acid concentrations could be achieved, and to preliminarily assess any response to treatment. In the nine patients who completed the study, ascorbic acid concentrations were reached safely and with minimal associated adverse events that could be attributed to ascorbic acid. Overall, the safety data do not reveal adverse events other than what might be expected for progression of pancreatic cancer and/or treatment with gemcitabine and erlotinib. Deaths of three patients who died before completing the study were attributable to underlying and rapidly advancing disease, as affirmed by the Data Safety and Monitoring Board.

Peak ascorbic acid concentrations were achieved as high as 30 millimoles/L in the highest dose group. These concentrations are similar to reported concentrations in patients who received ascorbic acid intravenously without concomitant chemotherapy [20]. For a frame of reference, the usual plasma ascorbic acid concentrations in people are 0.010–0.080 millimoles/L and are dependent on dietary and supplement intake. Even with massive oral supplementation of many grams daily taken every few hours, plasma ascorbic acid concentrations in people do not exceed 0.25 millimoles/L [13]. The data from this trial indicate that pharmacologic ascorbic acid concentrations were achievable in patients who received intravenous ascorbic acid in combination with gemcitabine and erlotinib.

CT images at the beginning and end of 8 weeks of treatment revealed that primary tumor size (target lesion) decreased in 8 of 9 subjects; was stable in the one subject who did not have a decrease; and specifically decreased in the three subjects who received the highest ascorbic acid dose (see Table 3 and Figure 3). Clinically, these findings are not typical with treatment using gemcitabine alone or with gemcitabine plus erlotinib [26], [27], [28], [29], [30], [31].

The behavior of non-target lesions also was concordant. In the highest ascorbate dose group, non-target lesions were either improved or stable, and 7 of 9 patients who had pre and post treatment CT scan evaluations had stable or improved non target lesions. However, since 3 additional patients died from rapid progression of the disease the overall result would suggest that 7 of 12 patients had stable disease. The data are consistent with observed synergy between gemcitabine and pharmacologic ascorbate in cell and animal experiments [20].

It is noted that RECIST 1.0 criteria for stable disease are inclusive of a 19% *increase* in target lesions [24]. Other studies of gemcitabine efficacy in pancreatic cancer that categorize disease as stable do not provide details concerning target lesion increases under 20%, meaning that subjects with target lesion size increases up to 19% are still considered stable disease. Therefore, the importance of our finding of target size *decrease* in 8 of 9 subjects may be underestimated. RECIST 1.0 criteria for partial response require that there be at least a 30% decrease in target lesions without definitive increase in non-target lesions. In addition, many efficacy studies of gemcitabine based therapies for pancreatic cancer patients include both metastatic and locally advanced patients [26]. Our study included only metastatic patients.

There were other potential issues with properly capturing the efficacy signal of ascorbic acid plus gemcitabine and erlotinib in this trial design. Ascorbic acid may act differently than classic cytotoxic chemotherapy. In particular, unlike many cancer therapies, ascorbate does not appear to have toxicity on rapidly dividing normal cells such as those in intestine cells, hair follicle cells, and bone marrow. Because of the absence of apparent tissue toxicity, effects of ascorbic acid treatment on human tumors might be expected to be more gradual, and as a corollary to require longer treatment. This possibility is consistent with observations from case reports of patients who received intravenous ascorbic acid as treatment for several types of cancers [20], [32], [33].

Given the possibility that longer ascorbic acid treatment is necessary to see disease improvement by RECIST 1.0 criteria, and the somewhat encouraging findings in the nine subjects in this trial, studying a longer treatment period at the 100 gram dosage seems warranted. Although our determination of progression free survival and overall survival were comparable to values previously reported for gemcitabine/erlotinib therapy alone [34], the data are limited by the short treatment duration with ascorbate. Our primary goal was to evaluate safety of the combination treatment and provide a preliminary assessment of treatment effect. Because ascorbic acid appears to be safe with concomitant gemcitabine and erlotinib, a next reasonable step would be a phase II study with patients randomized to ascorbic acid plus gemcitabine/erlotinib versus gemcitabine/erlotinib alone for a longer treatment duration and to assess for progression free and overall survival.

## Supporting Information

Protocol S1 Trial Protocol.
(PDF)

Checklist S1 CONSORT Checklist.
(DOC)
