# Supplementary material for: Phase I Evaluation of Intravenous Ascorbic Acid in Combination with Gemcitabine and Erlotinib in Patients with Metastatic Pancreatic Cancer
Source: PLoS One. 2012 Jan 17;7(1):e29794. doi: 10.1371/journal.pone.0029794 (PMC3260161; doi:10.1371/journal.pone.0029794)
Supplement: Protocol S1 — Trial Protocol. (PDF) [file pone.0029794.s001.pdf]

Phase I and pilot phase II, open label, dose escalating study of intravenous ascorbic acid in combination with gemcitabine and erlotinib in the treatment of metastatic pancreatic cancer. (Study limited to Phase I)

Submitted by: Daniel Monti, M.D. (PI, Protocol Oversight), Edith Mitchell, M.D. (Study Oncologist), Anthony Bazzan, M.D. (Study Physician)

Index

1.0 Objectives

2.0 Background and Rationale

3.0 Experimental Plan

4.0 Subject Eligibility

5.0 Methods

6.0 Study Parameters

7.0 Statistical Considerations

8.0 References

9.0 Appendix

1.0 OBJECTIVES

This is a Phase I, open label, limited dose escalation study of ascorbic acid, combined with gemcitabine and erlotinib in metastatic pancreatic cancer patients undergoing front-line chemotherapy.

1.1 Primary Aims

Determine safety and tolerability of combining intravenous (IV) Ascorbic Acid (AA) with gemcitabine and erlotinib therapy. This will be accomplished by enrolling 9-18 subjects meeting inclusion criteria, using a modified 3+3 design. Safety will be assessed with the following assessments: toxicity graded by the NCI CTC, basic metabolic panel, CBC, 24 hour urinalysis for uric acid, oxalate and osmolality pre- and post-eight week IVAA infusion cycle, and ECG.

1.2 Secondary Aims

To estimate:

- Blood levels of IV AA at study dosages
- Overall response rate

1.3 The study will be conducted under FDA IND #77,486.

This Phase I safety trial is an essential prerequisite to a phase II efficacy study to evaluate whether IV AA combined with Gemcitabine and erlotinib is beneficial. From our experience and in the experience and unpublished data of Drisko and colleagues at the University of Kansas (who have permitted us to cross reference their IND# 65,805, which has specific indications for IV AA in Pancreatic cancer) we hypothesize that the combination of IV AA with gemcitabine and erlotinib will not be associated with adverse events.

2.0 BACKGROUND AND RATIONALE

2.1 Pancreatic Adenocarcinoma

Pancreatic adenocarcinoma is the fourth-leading cause of cancer-related deaths in the United States (ACS, 2006), representing 6% of all cancer-related deaths. The disease appears to affect both males and females equally; however, it is most prevalent among those ages 65 and older and in African Americans (Jemal, 2006). The median survival of subjects with this disease is approximately 9 months, a statistic which makes pancreatic carcinoma an aggressive cancer. The lethality of this malignancy is demonstrated by the fact that the incidence of 33,730 is approximately equal to the annual deaths of 32,300 (ACS,2006).

Surgery is currently the only effective curative treatment; however due to late symptomatic presentation, <10% of patients diagnosed with pancreas cancer can actually have a curative resection (Yeo, 1997). With this intervention, the actuarial 5-year survival rates for these patients remains at approximately 20%, indicating that even patients with localized small cancers (< 2cm) with no lymph node metastases are likely to die of metastatic disease. Thus, the majority of subjects present with advanced unresectable cancer. This stage of disease has a 1-year survival of ~ 35% and a 5-year survival rate of only 3 to 4% (McKenna, 2003).

Current Treatment of Advanced Pancreatic Cancer

Since the approval of gemcitabine by the FDA in 1996, gemcitabine remains the leading treatment option for patients with advanced pancreatic cancer (NCCN, 2008). Although advanced disease is generally refractory to cytotoxic agents, single agent gemcitabine proved to have a clinical benefit response rate of 23.8% in this subject population, which is substantially better than that reported for 5-fluorouracil (5-FU) (4.8%) in subjects with advanced pancreatic cancer (Burris, 1997).

Combining gemcitabine with other cytotoxic chemotherapy agents has been largely unsuccessful. However, the NCIC-CTG PA3 trial demonstrated that the combination of gemcitabine with erotinib resulted in a statistically significant improvement in overall survival when compared to gemcitabine and placebo (hazard ratio 0.81, p=0.025; Moore et al, 2005). Median and one-year survival in the gemcitabine plus erlotinib arm were 6.4 months and 24%, respectively compared to 5.9 months and 17% in the gemcitabine plus placebo arm. Based on these results, the Food and Drug Administration (FDA) and the European Medicines Agency (EMEA) recently approved this regimen for the treatment of advanced pancreatic cancer. Clearly there is a room for additional therapies to treat this disease.

2.2 Rationale for Combining Gemcitabine and Erlotinib with Ascorbic Acid

Intravenous, high dose ascorbic acid is a widely used alternative cancer treatment, although its use as such has not been appropriately tested. Ascorbic acid as a cancer therapy was largely discarded several years ago when two randomized trials of oral vitamin C therapy failed to demonstrate therapeutic benefit (Creagan, 1979, Moertal, 1985). However, recent pharmacokinetic modelling indicates that intravenous administration of Vitamin C produces a 25-fold or greater plasma concentration than the same dose given orally (Padayatty, 2004). Chen and associates have reported that vitamin C levels achievable in vivo only by intravenous infusion are selectively cytotoxic in vitro to various cancer cell lines but not to normal cells by a mechanism involving formation of hydrogen peroxide (Chen, 2005). This mechanism is dependent on pro-oxidant actions, as a consequence of ascorbate concentrations achieved only by intravenous administration. Ascorbic acid is always chemically a reducing agent (anti-oxidant), or electron donor. However, at pharmacologic concentrations, ascorbate electron donation sets in motion a series of chemical reactions whose end result is formation of hydrogen peroxide and subsequent pro-oxidant compounds, with selective toxicity to cancer but not normal cells. Thus, while ascorbic acid action is always as an electron donor, its actions at pharmacologic concentrations produce pro-oxidant consequences. The mechanism of action of Vitamin C as a prodrug for hydrogen peroxide formation in the extravascular space has recently

been confirmed by the laboratory of study consultant Mark Levine, M.D. (Chen, 2007). This action of IV Vitamin C is consistent with a growing literature that reactive oxygen species play an important role in the mechanism of action of proven cancer treatments and that impaired oxygen-reduction balance in cancer cells might cause induced reactive oxygen species to selectively kill cancer cells (Chen, 2007).

A recent report found three well-documented cases of advanced cancers, confirmed by histopathologic review, where patients had unexpectedly long survival times after receiving high-dose intravenous vitamin C therapy (Padayatty, 2006). The report examined clinical details of each case in accordance with National Cancer Institute (NCI) Best Case Series guidelines, and tumour pathology was verified by pathologists at the NCI who were unaware of diagnosis or treatment.

Levine's group just published a study on tumor xenographs in mice confirming ascorbic acid as a prooxidant that decreased growth of three aggressive tumor types: Pancreatic, glioblastoma, and ovarian (Chen, 2008). To test this action *in vivo*, the investigators bypassed normal oral tight control with parenteral ascorbate administration. Real-time microdialysis sampling in mice bearing glioblastoma xenografts showed that a single pharmacologic dose of ascorbate produced sustained ascorbate radical and hydrogen peroxide formation selectively within interstitial fluids of tumors but not in blood. Moreover, a regimen of daily pharmacologic ascorbate treatment significantly decreased growth rates of ovarian ($P < 0.005$), pancreatic ($P < 0.05$), and glioblastoma ($P < 0.001$) tumors established in mice. Also in this study, similar pharmacologic concentrations were readily achieved in humans given ascorbate intravenously. These data suggest that ascorbate as a prodrug may have benefits for some cancers. Overall, recent in vitro biological evidence, limited human case data, clinical pharmacokinetic data, and recent in vivo data confer biological *plausibility* to the notion that vitamin C could affect cancer biology and that there is reason to explore the therapeutic concept (see fig. 1). More research data is needed to guide the current wide use of high dose intravenous vitamin C.

A recent report in the literature suggested that the anti-oxidant affects of Vitamin C could interfere with chemotherapeutic efficacy (Heany et al, 2008). We note that this report used a mouse model of Dihydro-Ascorbic Acid (DHA), which is chemically different from ascorbic acid and would never be used in human models because of its toxicity. Moreover, the authors of that study theorized that an anti-oxidant effect may have provided cancer cell production. We underscore that the dosages of IV AA proposed in the present study are such that the net effect is clearly *pro-oxidant,* as described above.

Due to the poor outcome of subjects with surgically unresectable metastatic pancreatic cancer, there is a need for further investigational studies in the disease management and treatment of this cancer. Gemcitabine monotherapy or gemcitabine combined with erlotinib, remain the leading treatment options for patients with surgically unresectable or metastatic pancreatic cancer. We propose a phase I / II single agent study of intravenous Vitamin C to measure the activity and toxicity profile of this agent in patients with metastatic pancreatic cancer also receiving gemcitabine and erlotinib.

Fig. 1 Proposed ascorbate mechanism of action schematic:

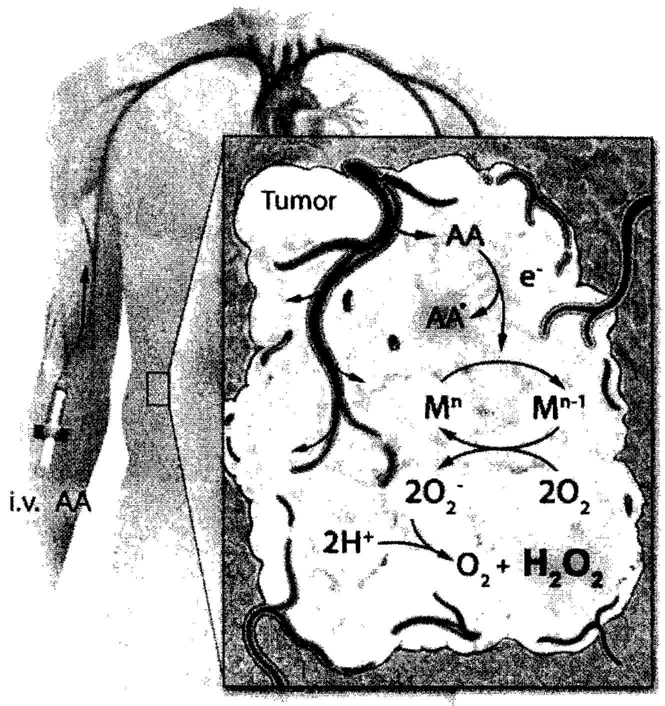

2.3 Ascorbic Acid Toxicity

Ascorbic acid has been shown to be well tolerated when administered intravenously at high doses (Chen, 2005), and phase I human data from a group of mixed cancer patients confirm it to be relatively safe and non-toxic, even at doses as high as 1.5 grams/Kg (Hoffer et at 2008). Two cases of acute oxalate nephropathy were reported in patients with pre-existing renal insufficiency given massive intravenous doses of vitamin C (Wong et al, 1994); therefore, patients with renal insufficiency or renal failure, or who are undergoing dialysis, should not receive high doses of vitamin C. There are two reports of intravascular hemolysis in people infused with more than 50 g ascorbic acid and one that describes cases of hemolysis in persons who received at least 6 g of ascorbic acid as a single oral dose. All of the cases were in persons with the rare congenital metabolic disease, glucose-6-phosphate dehydrogenase (G6PD) deficiency, which renders red blood cells susceptible to oxidative stress (Riordan et al, 2004). G6PD status will be evaluated prior to start of therapy for all potential subjects.

2.4 Hypotheses

The primary hypothesis for the phase I study is that combining gemcitabine and erlotinib with IV AA will not be associated with serious adverse affects. Blood levels of IV AA at the three study dosages will also be assessed.

2.5 Preliminary Studies (conducted by Drisko and colleagues, FDA IND # 65,805; we cross-reference this IND with written permission and Dr. Drisko is a consultant to the proposed study)

2.5.1 Case Reports

Drisko and collaborators have reported on 2 cases of advanced ovarian cancer that were treated with high dose intravenous AA alongside chemotherapy (Drisko et al., 2003). Both patients continue to receive intravenous ascorbate infusions at the time of this writing on an every other week schedule at a level to optimize plasma AA levels. Both patients are free of recurrence at over 5 years with normal tumor markers and are seen on a regular basis by either the investigators or their associates.

2.5.2 Drisko et al ongoing randomized controlled clinical trial

The antioxidant-ovarian cancer study is a prospective randomized phase II study comparing standard of care for advanced ovarian cancer to standard of care combined with intravenous and oral antioxidant therapies. Study subject population consists of newly diagnosed ovarian cancer Stage III or IV with optimal or suboptimal debulking at staging laporotomy. Patients are all required to have at least six cycles of carboplatin and paclitaxel chemotherapy with care provided at one of two registered sites and therapy dictated by the co-investigators in this trial. Antioxidant and intravenous AA are administered for 12 months and then discontinued. The trial is closed to enrollment. Dr Drisko is assisted in this study by Dr Julia Chapman, Division Chief GYN Oncology and by Dr Stephen Williamson, Director of Medical Oncology at the University of Kansas Medical Center.

Descriptions of toxicities evaluated during interim safety analysis in early 2005 are summarized in tables below and show highest toxicity per subject in both arms. Adverse events were evaluated by the National Cancer Institute Common Terminology Criteria for Adverse Events v3.0 (CTCAE) and scored from 1 through 5 with 3 to 5 considered significant adverse events. Adverse events most commonly occurred during administration of chemotherapy and were categorized by the NCI CTCAE classification; the most common adverse events recorded were related to Blood/Bone Marrow Toxicities and Neurology Toxicities – Neuropathy.

More than 800 doses of intravenous ascorbic acid were given to the participants in the treatment arm at the time of this interim safety analysis, both with and without co-administered chemotherapy. The majority of doses were at 75 or 100 grams per infusion. Overall there does not appear to be a disproportionate number of adverse events in the treatment arm when compared to the control arm, although it is possible that the administration of chemotherapy may be masking some events that could be attributed to IV ascorbic acid. Since the numbers do not allow evaluation of statistical significance at this time, we are simply reporting events in tabular form.

Table 1 Highest Toxicity Grades across **All Categories summarizing all Events**

Group	1	2	3	4	5	None
**Control** N (%)	0 (0%)	4 (30.8%)	7 (53.8%)	1 (7.7%)	0 (0%)	1 (7.7%)
**Treatment** N (%)	0 (0%)	0 (0%)	5 (41.7%)	4 (33.3%)	0 (0%)	3 (25.0%)

Table 2 Summary of All **Blood/Bone Marrow Toxicities** Inclusive of WBC, RBC, and Plts

Group	1	2	3	4	5	None
**Control** N (%)	2 (15.4%)	4 (30.8%)	6 (46.2%)	0 (0%)	0 (0%)	1 (7.7%)
**Treatment** N (%)	0 (0%)	1 (8.3%)	4 (33.3%)	4 (33.3%)	0 (0%)	3 (25.0%)

Table 3 Summary of Toxicities across All **Renal / Genitourinary Categories**

Group	1	2	3	4	5	None
**Control** N (%)	1 (7.7%)	1 (7.7%)	0 (0%)	0 (0%)	0 (0%)	11 (84.6%)
**Treatment** N (%)	0 (0%)	0 (0%)	0 (0%)	0 (0%)	0 (0%)	12 (100.0%)

Table 4 Summary of across All **Pulmonary Toxicities**

Group	1	2	3	4	5	None
**Control** N (%)	0 (0%)	2 (15.4%)	0 (0%)	0 (0%)	0 (0%)	11 (84.6%)
**Treatment** N (%)	0 (0%)	0 (0%)	0 (0%)	0 (0%)	0 (0%)	12 (100.0%)

Table 5 Summary of Toxicities across all **Gastrointestinal Categories** – Including Nausea, Constipation, Incontinence, Esophagitis, Pain, Obstruction

Group	1	2	3	4	5	None
**Control** N (%)	2 (15.4%)	5 (38.4%)	2 (15.4%)	0 (0%)	0 (0%)	4 (30.8%)
**Treatment** N (%)	0 (0%)	2 (16.7%)	4 (33.3%)	0 (0%)	0 (0%)	6 (50.0%)

Table 6. Highest Toxicity Grade Across **All Infection Categories**

Group	1	2	3	4	5	None
**Control** N (%)	0 (0%)	4 (30.8%)	0 (0%)	0 (0%)	0 (0%)	9 (69.2%)
**Treatment** N (%)	0 (0%)	2 (16.7%)	0 (0%)	0 (0%)	0 (0%)	10 (83.3%)

Table 7. Highest Toxicity Grade Across **All Hepatobiliary/Pancreas Categories**

Group	1	2	3	4	5	None
**Control** N (%)	0 (0%)	0 (0%)	0 (0%)	0 (0%)	0 (0%)	13 (100.0%)
**Treatment** N (%)	0 (0%)	2 (16.7%)	0 (0%)	0 (0%)	0 (0%)	10 (83.3%)

Table 8. Highest Toxicity Grade Across **All Neurology Categories**

Group	1	2	3	4	5	None

**Control** N (%)	4 (30.7%)	5 (38.5%)	0 (0%)	1 (7.7%)	0 (0%)	3 (23.1%)
**Treatment** N (%)	3 (25%)	1 (8.3%)	2 (16.7%)	0 (0%)	0 (0%)	6 (50.0%)

2.5.3 Current Patient Receiving IV Ascorbic Acid for Stage IV Pancreatic Cancer

The patient is a 73 year old female diagnosed with adenocarcinoma of the head of the pancreas on 9/03/2006. At diagnosis, the mass was 5 cm with metastatic disease to retroperitoneal nodes and extension of the mass into the hilum of the liver with obstruction of the biliary tree. The patient was Stage IV at diagnosis and ineligible for surgical resection. Biliary stents were placed to decompress the biliary tree and chemotherapy was proposed by her treating oncologist. Patient elected to begin intravenous ascorbic acid prior to starting chemotherapy. She currently receives intravenous ascorbic acid on a 3 times per week schedule at a dose of 125 grams per infusion (a dosage that is slightly higher than what is requested as a ceiling dose for this protocol). The patient also receives gemcitabine (Gemsar 1,000 mg/m2 on days 1 and 8) and erlotinib (Tarceva 100mg tab/day) chemotherapy. The patient's performance status is ECOG 0 with only minimal complaints of pain, excellent quality of life and no evidence of weight loss. Currently at 10-months post diagnosis, her most recent CT scan remains stable with no evidence of progression of disease. While this is an anecdotal case suggesting benefit when adding ascorbic acid infusions to the treatment of pancreatic cancer, it reflects our previous positive experience in treating this difficult neoplasm and warrants further investigation.

3.0 EXPERIMENTAL PLAN

3.1 Study Design

This is a phase I open-label, dose escalating clinical trial to be conducted at Thomas Jefferson University Hospital. Nine to eighteen metastatic pancreatic cancer patients will be invited to participate in this phase I study.

Requirements for clinical and radiological assessments are outlined in Section 6.0.

All subjects meeting eligibility criteria will be enrolled into the study to receive gemcitabine and IV AA. Gemcitabine will be administered intravenously at a dose of 1000 mg/m^2 over 30 minutes, once weekly for 7 weeks followed by a 1 week rest. Subsequent cycles consist of injections,once weekly for 3 consecutive weeks out of every 4 weeks,but may be modified by the medical oncologist co-Investigator. Erlotinib will be given orally in a single dose of 100mg per day for the first cycle and then determined by the medical oncologist co-Investigator.

In this phase I study, IV AA therapy will occur in nine to eighteen subjects across three planned escalations. The first cohort will receive 50 grams IVAA. A cycle will consist of three infusions per week with at least 24 hours between infusions, for 8 weeks (twenty-four infusions). Enrollment into the next cohort cannot begin until 1 week has elapsed since the last subject's infusion during the last week of their cyclein the previous cohort. The study is a standard 3+3 design with only 3 Dosages planned (50 gm/infusion; 75 gm/infusion; 100 gm/infusion). It is noted that each cohort will start at the 50 gm/infusion dosage and titrate to 75gm/infusion on

infusion 2 for those in cohort 2, and for those in cohort 3, there will be a second titration on the third infusion to 100 grams/infusion. AA blood levels will be drawn immediately following the infusion of a new dosage. 100 grams per infusion is the arbitrary ceiling dosage for this study, primarily because that dosage usually reflects a blood level that is currently felt to be high enough to achieve the proposed mechanism of action (Jackson et al, 1995) and it is also the ceiling dosage for the primary study of this IND. In addition, 100 gm/infusion of AA is the ceiling dosage allowed at Thomas Jefferson University Hospital for those receiving AA supplementation and not on a study protocol. Fifty grams is chosen as the starting dosage based upon its established safety in other studies (Hoffer et al, 2008).

As described later, chemotherapy with gemcitabine/erlotinib and IV AA will be administered until one of the following occurs: disease progression, unacceptable adverse events, death or withdrawal from study by the subject, investigator, or sponsor. Following gemcitabine/ erlotinib and IV AA discontinuation and a safety visit, 30 days ±3 days after completion of treatment (last dose), has occurred, subjects will be followed every month for survival.

3.2 Number of Subjects

Participants of this clinical investigation shall be referred to as "subjects." The planned sample size is 9-18 subjects for this phase I study.

3.3 Estimated Study Duration

The maximum study duration is expected to be 24 months. The expected duration of subject accrual is 12 months. The maximum duration of treatment is estimated to be approximately 9 months and long-term follow-up will be 12 months from the date of last subject's enrollment. Subjects will be treated on study until one of the following occurs: disease progression, unacceptable adverse events, death or withdrawal from study by the subject or investigator.

3.4 End of Study

End of study for a subject is defined as:

- Completion of the post-treatment follow-up phase (12 months after the last subject is enrolled), death, lost-to-follow-up, or study withdrawal by the subject (fully withdrawn informed consent), investigator, or sponsor, whichever occurs first

- For ongoing subjects who continue to receive treatment beyond 12 months after the last subject is enrolled, completion of the post-treatment follow-up will occur upon unacceptable adverse events(s), disease progression, death, lost-to-follow-up, or study withdrawal by the subject (fully withdrawn informed consent), investigator, or sponsor, whichever occurs first

End of Study is defined as discontinuation of the last ongoing subject due to unacceptable adverse events, disease progression, death, lost-to-follow-up, or study withdrawal by the subject, investigator, or sponsor, whichever occurs first.

4.0 SUBJECT ELIGIBILITY

Before any study-specific procedures are performed, all subjects screened for participation must voluntarily sign and date the appropriate informed consent form approved by the Thomas Jefferson University institutional review board (IRB).

Investigators will be expected to maintain a screening log of all potential study candidates that includes limited information about the potential candidate (eg, age, gender, and race), date, and outcome of the screening process (eg, enrolled into the study, reason for ineligibility, or refused to participate).

4.1 Inclusion Criteria

The following criteria must be met at the time of subject enrollment:

4.1.1 Demographic

- Males and females ≥18 years of age

4.1.2 Disease related

- Histologically or cytologically confirmed pancreatic adenocarcinoma
- Metastatic Disease that is measurable by CT or MRI
- Subjects with unresectable pancreatic cancer who have had surgery (exploratory laparotomy, bilary, gastrointestinal bypass) are eligible, if the subject has fully recovered from surgery and ≥ 28 days has passed since the operation. Patients with history of pancreatoduodenectomy are eligible provided that there is radiographically documented disease recurrence.
- ECOG performance status 0-2
- Life expectancy of ≥ 12 weeks as documents by the study oncologist

4.1.3 Laboratory

- Hematologic function, as follows:
  - Absolute neutrophil count (ANC) ≥1.5 x 10^9/L
  - Platelet count ≥100 x 10^9/L
  - Hemoglobin ≥9.0 g/dL
  - G6PD status normal
- Renal function, as follows:

- Serum creatinine ≤1.5 mg/dL

- Hepatic function, as follows:

  - Aspartate aminotransferase (AST) ≤ 3 x ULN (if liver metastases ≤ 5 x ULN)
  - Alanine aminoransferase (ALT) ≤ 3 x ULN (if liver metastases ≤ 5 x ULN)
  - Total bilirubin ≤ 1.2 mg/dL. Patients with history of biliary obstruction are eligible after intervention, once this criteria is met

- Metabolic function, as follows:

  - Magnesium ≥ lower limit of normal
  - Calcium ≥ lower limit of normal

4.1.4 General

- Competent to comprehend, sign, and date an IRB-approved informed consent form

4.2 Exclusion Criteria

If the subject meets any of the following criteria at the time of enrollment, he or she will not be eligible for this trial:

4.2.1 Disease related

- Islet cell or acinar cell carcinoma or cystadenocarcinoma

History or known presence of central nervous system (CNS) metastases

- History of another primary cancer, except:

  - Curatively treated cervical carcinoma in situ, or

  - Curatively resected non-melanomatous skin cancer, or

  - Other primary solid tumor curatively treated with no known active disease present and no treatment administered for ≥ 3 years prior to enrollment

- Other concurrent anticancer chemotherapy

- Concomitant malignant disease

- Prior radiotherapy ≤ 14 days, or if subjects have not recovered from radiotherapy

- Uncontrolled seizure disorder or other serious neurological diseases

- Any co-morbid disease that would increase risk of toxicity

- Only locally advanced disease

4.2.2 Medications/Treatments

- Adjuvant chemotherapy or chemoradiotherapy
- Prior treatment with gemcitabine for metastatic disease within the previous 12 months
- Subjects requiring chronic use of immunosuppressive agents (eg, methotrexate, cyclosporine, corticosteroids)
- Regular use (as determined by the investigator) of nonsteroidal anti-inflammatory agents
- Recent infection requiring a course of systemic anti-infectives that was completed ≤ 14 days prior to enrollment (exception can be made at the judgment of the investigator for oral treatment of an uncomplicated urinary tract infection ([UTI])

4.2.3 General

- Clinically significant cardiovascular disease (including myocardial infarction, unstable angina, symptomatic congestive heart failure, serious uncontrolled cardiac arrhythmia) ≤ 1 year prior to enrollment
- History if interstitial lung disease (eg, pneumonitis or pulmonary fibrosis or evidence of interstitial lung disease) on screening chest x-ray or CT scan
- Pulmonary embolism, deep vein thrombosis, or other significant thromboembolic event ≤ 8 weeks prior to enrollment
- Pre-existing bleeding diathesis or coagulopathy with the exception of well-controlled chronic anticoagulation (eg, coumadin or heparin therapy). Subjects receiving coumadin should have their INR monitored closely.
- G6PD negative: Red blood cell hemolysis from high dose AA may occur in people found to be deficient in the G6PD enzyme.
- History of any medical or psychiatric condition or addictive disorder, or laboratory abnormality that, in the opinion of the investigator, may increase the risks associated with study participation or study drug administration or may interfere with the conduct of the study or interpretation of study requirements
- Subject unwilling or unable to comply with study requirements
- Subject who is pregnant or breast feeding

- Man or woman of child-bearing potential (women who are post-menopausal < 52 weeks, not surgically sterilized, or not abstinent) who do not consent to use adequate contraceptive precautions (per institutional standard of care) during the course of the study and for 24 weeks for women and 4 weeks for men, after the last dose of gemcitabine or IV AA, whichever dose is last

- Known positive test(s) for human immunodeficiency virus infection, hepatitis C virus, chronic active hepatitis B infection

- Major surgery ≤ 28 days or minor surgery ≤ 14 days prior to enrollment

- Documented history of alcohol, cocaine or intravenous drug abuse ≤ 6 months of enrollment

4.3 Subject Enrollment

Medical history collected during screening must go back to the initial diagnosis of pancreatic cancer. If subjects are referred to the study center, a copy of all applicable reports and histological or cytological evidence confirming the diagnosis must be provided to the study center before enrollment. Copies of recent (ie, within the past 21 days) radiographic images confirming disease sites should be provided to the study center.

All subjects who enter into the screening period of the study (defined as the point at which the subject signs the IRB approved informed consent) will receive a unique subject identification number before any study procedures are performed. This number will be used to identify the subject throughout the clinical study and must be used on all study documentation related to that subject.

5.0 METHODS

5.1 Description of Investigational Intervention

5.1.1 Structure and Molecular Weight:

Ascorbic acid is a 6-carbon ketolactone structurally related to glucose and other hexoses. Its formal chemical structure is 3-oxo-L-gulofuranolactone, $C_6H_8O_6$ © 40.9%, H 4.6% O 54.5%), molecular weight 176.13.

5.1.2 Investigational Formulation and Supplier:

Ascorbic Acid Injection USP is provided by American Regent Inc., Shirley, New York. It is ordered through the Jefferson pharmacy. It is a clear, colourless to slightly yellow sterile solution of ascorbic acid in water for injection, for intravenous, intramuscular, or subcutaneous use. Each mL contains ascorbic acid 500 mg (2.84 mmol), edetate disodium 0.025%, and water for injection q.s. with the pH (range 5.5 to 7.0) adjusted with sodium bicarbonate, hence

providing ~ 2.84 mmol sodium. The product is supplied in sterile 50 mL single use glass ampuls containing 500 mg/mL of ascorbic acid USP. The stock solution contains 2.84 mmol of ascorbic acid per mL and 2.84 mmol of sodium per mL for a theoretical osmolality of 2.84 + 2.84 = 5.7 mOsm/mL.

5.1.3 Storage and Preparation of Material:

The product is stored in a carton protected from light between 2 - 8 °. C. It must not be frozen. It must be infused within 4 hours of preparing diluted solutions for injection. Doses less than 15 g the dose are mixed with Ringers lactate to guarantee a sufficiently high osmolality. When the dose is equal to or greater than 15 g, it is brought to the required concentration in sterile water to achieve an osmolality between 500 and 800 mOsm/L. This can be calculated from the molecular weight of ascorbic acid with the assumption that ascorbic acid is completely dissociated in solution. Examples are given as follows:

- 15 g (30 mL stock brought to 300 mL using sterile water): 570 mOsm/L, total sodium: 85 mmol.
- 30 g (60 mL stock brought to 500 mL using water): 684 mOsm/L, total sodium 170 mmol.
- 60 g (120 mL stock brought to 900 mL using water): 760 mOsml/L, total sodium 340 mmol.
- Infusion Carrier Fluid: IV infusions will be administered in sterile water or lactated Ringers (see Table 1) depending on the amount of ascorbic acid and other nutrients to be infused. Magnesium chloride is also added to the solutions of 25 grams or higher ascorbic acid. See Table 1 bolded numbers for correct carrier solutions

Sodium ascorbate/Ascorbic	Final Volume of Sterile water				Final Volume of Ringer's Lactate			
Acid (g)	250	500	750	1000	250	500	750	1000
1	39	19	13	10	**336**	**318**	**312**	**309**
15	**579**	290	193	145	**843**	**572**	**481**	**436**
30	1158	**579**	**386**	290	1386	**843**	**662**	**572**
60	2316	**1158**	**772**	**579**	2472	1386	**1024**	**843**
75	2895	1448	**965**	**724**	3015	1658	1205	**979**
100	3860	1930	1287	**965**	3920	2110	1507	*1205*

5.2 Gemcitabine

5.2.1 Other Names/Classification

2' -Deoxy- 2', 2' -difluorocytidine monohydrochloride, Gemzar / Antimetabolite (nucleoside analog)

5.2.2 Mode of Action

Gemcitabine exhibits cell phase specificity, primarily killing cells undergoing DNA synthesis (S phase) and also blocking the progression of cells through the Gl/S phase boundary. Gemcitabine is metabolized intracellularly by nucleoside kinases to the active diphosphate (dFdCDP) and triphosphate (dFdCTP) nucleosides. Gemcitabine triphosphate competes with dCTP for incorporation into DNA. The reduction in the intracellular concentration of dCTP (by the action of the diphosphate) enhances the incorporation of gemcitabine triphosphate in DNA (selfpotentiation). After the gemcitabine nucleotide is incorporated into DNA, only one additional nucleotide is added to the growing DNA strands. After this addition, there is inhibition of further DNA synthesis.

5.2.3 Storage and Stability

Unreconstituted drug vials are stored at controlled room temperature. Reconstituted solution should be stored at controlled room temperature and used within 24 hours. Solutions of gemcitabine should not be refrigerated; crystallization may occur. The unused portion should be discarded.

5.2.4 Dose Specifics

Dosage calculations will be based on actual, rather than ideal, body weight. When ascites is present, use estimated "dry" body weight.

- The first cycle consists of gemcitabine at 1000 mg/m² as a 30 minute IV infusion once a week for 7 weeks followed by one week rest. Thereafter patients receive gemcitabine at 1000 mg/m² IV over 30 minutes once a week for 3 weeks followed by one week rest until progression.

5.2.5 Preparation

Reconstitute the 200 mg vial with 5 ml and the 1 gm vial with 25 ml preservative free normal saline to make a solution containing 38 mg/ml. Shake to dissolve.

5.2.6 Administration

Gemcitabine is given intravenously as a 30 minute infusion. The drug may be administered as prepared or further diluted with normal saline to a minimum concentration of 0.1 mg/ml. Gemcitabine is commonly diluted in 100 ml or 250 ml of saline.

5.2.7 Incompatibilites

No information available.

5.2.8 Availability

Gemcitabine is commercially available in 200 mg and 1 gm vials.

5.2.9 Side Effects

Hematologic: Myelosuppression is usually mild to moderate and is more pronounced for the granulocyte count. Thrombocytopenia is also commonly reported.

Dermatologic: A rash is seen in about 25% of patients and is associated with pruritus in about 10% of patients. The rash is usually mild, not dose-limiting, and responds to local therapy. Desquamation, vesiculation, and ulceration have been reported rarely. Alopecia is reported in <1% of patients.

Gastrointestinal: Nausea and vomiting are reported in about two-thirds of patients and require therapy in about 20% of patients. It is rarely (<1%) dose-limiting, and is easily managed with standard antiemetics. Diarrhea is reported in 8% of patients, constipation in 6%, and oral toxicity in 7%.

Hepatic: Abnormalities of hepatic transaminase enzymes occur in two-thirds of patients, but they are usually mild, nonprogressive, and rarely necessitate stopping treatment. However, gemcitabine should be used with caution in patients with impaired hepatic function.

Pulmonary: Bronchospasm after injection has been reported in less than 1% of patients and is usually mild and transient, but parenteral therapy may be required. Dyspnea within a few hours of injection is reported in 10% of patients. It is usually mild, short-lived, rarely dose-limiting, usually abates without any specific therapy. Cough and rhinitis are also commonly reported. However, persistent respiratory symptoms, including shortness of breath, cough or pulmonary infiltrates, require additional evaluation. Fatal pulmonary complications have been associated with gemcitabine.

Neurologic: Somnolence has been reported in 10% of patients, and insomnia is common.

Cardiovascular: A few cases of hypotension were reported. Some cases of myocardial infarction, congestive heart failure, and arrhythmia have been reported; but there is no clear evidence that gemcitabine causes cardiac toxicity. Peripheral edema is reported in about 30% of patients. Some cases of facial edema have also been reported. Edema is usually mild to moderate, rarely dose-limiting, sometimes painful, and reversible after stopping gemcitabine treatment.

Other: Flu-like symptoms are reported for about 20% of patients. This includes fever, headache, back pain, chills, myalgia, asthenia, and anorexia. Malaise and sweating are also commonly reported.

5.2.10 Nursing/Patient Implications

If the patient reports burning at the injection site, hold until pain resolution. Wet compresses, warm or cool, may be used. Gemcitabine is not a vesicant.
Rash can be treated with topical therapy or the administration of diphenhydramine and dexamethasone prior to administration.
Flu-like symptoms can be treated with acetaminophen.

5.3 Erlotinib

5.3.1 Other Names/Classification

(CP-358 774; OSI-774/R1415; Tarceva®, OSI/Genentech),

5.3.2 Mode of Action

Erlotinib is an oral, human epidermal growth factor receptor type 1/epidermal growth factor receptor(HER1/EGFR) tyrosine kinase inhibitor. HER1/EGFR have been associated with increased tumor growth, proliferation, angiogenesis, metastasis, and inhibition of apoptosis. it is thought to reversibly bind to the adenosinetriphosphate (ATP) binding site of the tyrosine kinase domain associated with HER1/EGFR, located on the surface of normal and cancer cells.5 This binding inhibits the phosphorylation of the tyrosine kinase, interfering with cell communication, signal transduction, and, ultimately, cellular growth.

5.3.3 Storage and Stability

Commercial drug supply will be utilized and stored at room temperature.

5.3.4 Dose specifics

The dose will be 100 mg per day, taken orally with food.

5.3.5 Preparation

A thirty day supply of 100 mg tablets will be prescribed and refilled as needed.

5.3.6 Administration

The dose will be taken daily after a meal.

5.3.7 Incompatibilities

Erlotinib is metabolized primarily in the liver by the CYP3A4 enzyme. Coadministration with any CYP3A4 inhibitor (e.g., ketoconazole, ciprofloxacin, metronidazole, clarithromycin, or fluconazole) would consequently increase erlotinib serum levels. Conversely, coadministration with a CYP3A4 inducer (e.g., carbamazepine, phenytoin, rifampin, nafcillin) would probably decrease the serum concentration.of erlotinib. Patients requiring these medications will not be enrolled in the study.

5.2.8 Availability

Commercial resources will be used.

5.2.9 Side Effects

The most common ADEs reported in patients receiving erlotinib were rash and diarrhea. Grade 3 and 4 rash occurred in 9% of patients, and grade 3 or 4 diarrhea was reported in 6%.

- Rare cases of interstitial lung disease (0.6%) occurred between five days and nine months (median, 47 days) after the initiation of therapy.
- Asymptomatic elevations in liver function enzymes were observed.
- Increases in International Normalized Ratios (INRs), along with rare instances of bleeding, were reported.

5.4 Treatment Plan

5.4.1 Enrollment

- Informed consent will be obtained from all participants before protocol specific treatments are carried out. The participants will be informed about the nature of the study, its intended purpose, possible risks and benefits, controversies, and possible adverse events. Informed consent will be documented by use of written consent form approved by the Thomas Jefferson University Institutional Review Board.

- Participants will receive primary oncological care from study oncologist, Dr. Mitchell, or one of the medical oncology co-Investigators, who will determine eligibility and provide all required history and physical exam information for the study chart, which will be stored in confidential, secured file cabinet in the Jefferson-Myrna Brind Center of Integrative Medicine.

- Baseline Assessment Information for Documentation

  - Inclusion/Exclusion criteria – Patient eligibility
  - Demographics – date of birth, sex, race, and ethnicity
  - Relevant Medical History and Current Medical Conditions – related and unrelated to diagnosis
  - Physical Examination / Vital Signs – Baseline assessment present in the medical record
  - Hematology – Hemoglobin, total WBC, platelet count, differential,
  - Biochemistry – G6PD, BUN, creatinine, uric acid, albumin, total protein, bilirubin, alkaline phosphatase, AST (SGOT), ALT (SGPT), and LDH, and 24 hour urine analysis for oxalate and osmolality.
  - Comments – Any additional information will be captured in the study record in the comments section

5.4.2 Treatment Procedure

- History and physical examination and all laboratory analysis will be done within 14 days prior to registration and registration will occur prior to beginning the protocol.

- The first visit will be a screening visit (30 minutes) with review of medical records and laboratory work; timing of infusion visit(s) will be set on the initial visit

- Week-1, cohort 1 (phase I), will consist of 3 visits to the Brind Center to receive 3, 50 gram doses of IV AA. A plasma ascorbate level will be drawn immediately following the first infusion. Patients will be evaluated for toxicity and tolerability after each infusion.

- Week-1, cohort 2 (phase I), will consist of the same three visits as cohort 1 with the exception that the dosage of AA will be titrated on infusion 2 to 75 grams. The dosage will remain at 75 grams for the remainder of the cycle. A plasma ascorbate level will be drawn after the second infusion. Patients will be evaluated for toxicity and tolerability after each infusion.

Week-1, cohort 3 (phase I), will consist of the same three visits as cohort 2 with the exception that the third infusion will be titrated to 100 grams. The dosage will remain at 100 grams for the remainder of the cycle. A plasma ascorbate level will be drawn after the third infusion. Patients will be evaluated for toxicity and tolerability after each infusion.

- Week-2-8 for all three cohorts of this phase I study will consist of co- administration of gemcitabine at the Jefferson Kimmel Cancer Center's infusion center and three visits to the Kimmel Cancer Center's Brind Center to receive IV C; all IV AA infusions will be at least 24 hours apart. In addition, participants will be given erlotinib 100 mg. per day. The IV AA dosages in weeks 2-8 will be the same as dose given on day 3 of week 1.

  Gemcitabine will be administered intravenously at a dose of 1000 mg/m^2 over 30 minutes. Gemcitabine and erlotinib dosages may be adjusted if subsequent cycles of combination therapy are given.

5.4.3 Plasma Ascorbate Analysis

The plasma ascorbate analysis will be provided by our collaborator, Dr Mark Levine, at the National Institutes of Health, Chief of the Molecular and Clinical Nutrition Section, Digestive Diseases Branch, NIDDK. Blood will be collected in green top tubes on the prescribed schedule and will be immediately centrifuged at 1,000Xg for 10 minutes. Plasma is removed and frozen at -80°C and shipped on dry ice by overnight courier to the lab of Dr Levine. Plasma ascorbate levels will be measured by using high-performance liquid chromatography with coulometric electrochemical detection (Padayatty *et al.,* 2004): samples are stored on ice in a dark refrigerator until prepared for assay according to protocol. One volume of sample is mixed with 4 volume of 90% methanol/1mM EDTA in water and centrifuged in a refrigerated centrifuge at 25,000Xg. The supernatant is transferred to a new tube, immediately quick frozen in an acetone/dry ice bath, stored at -80°C until analyzed.

5.4.4 Off Study Procedure

Study subjects who complete the study or come off study will have a follow-up visit with laboratory analysis for hepatic and renal function

5.5 Criteria for Removal From / Cessation of Protocol

- Unacceptable toxicity – For individuals, unacceptable toxicity (per NCI Manual of Toxicities version 3.0) related to study drug as described below in section 5.6.
- Study subjects who are found to be using more than 1ppd of tobacco products will be removed from the protocol.
- The patient may withdraw from the study at any time for any reason.
- Missing 4 IV C infusions in a row or more than 6 infusions in one cycle.
- Completion of protocol
- All reasons for discontinuation of treatment will be documented in the flow sheets
- Protocol will be terminated at any point according to stopping rules in section: Human Subjects, Protection of Human Subjects / Potential Risks
- If more than 2 participants enrolled experience Grade 4 toxicities by CTC criteria, further enrollment will be stopped.

5.6 Toxicities to be Monitored

- Adverse events are evaluated by the National Cancer Institute Common Terminology Criteria for Adverse Events v3.0 (CTCAE) and scored from 1 through 5 with 4 to 5 considered significant adverse events.

- Adverse events, whether volunteered by the study subject, discovered by the investigators during questioning, or detected by physical examination, laboratory tests, or other means will be collected and recorded. An adverse event is an undesirable sign, symptom, or medical condition that occurs after the start of the study drug even if the event is not considered to be related to study drug. Medical conditions or disorders present before starting the study drug are considered adverse events if they worsen after starting study treatment. Adverse events starting after signing the consent but before starting the study drug will be recorded. Abnormal laboratory values or tests are considered adverse events. A serious adverse event is an undesirable sign, symptom, or medical condition which is fatal or life-threatening, requires prolonged hospitalization, results in significant or persistent disability/incapacity, is medically significant, may jeopardize the subject and may require medical or surgical intervention.

- Toxicities will be assessed using the NCI Common Toxicity Criteria (version 3.0). If at any time there are two instances of grade 4 toxicity referable to the study drug, further accrual will be halted. All data collected to that time point will be analyzed on an intent-to-treat basis.

- Patients experiencing Grade 4 neutropenia, Grade ≥3 thrombocytopenia, or Grade 2 peripheral neuropathy that do not recover will have treatment protocol discontinued.

- Neutropenic fever is defined as fever >101° in the face of a granulocyte count <1,000.

- Colony-stimulating factors will be prescribed at the discretion of the treating oncologist.

- Intravenous ascorbic acid in the doses administered in this study has been shown not to be toxic. To ensure that these therapies are non-toxic, liver enzymes and creatinine will be monitored bi-weekly during the treatment phase.
- Hepatic dysfunction attributable to therapy is defined as transaminase (AST/ALT) ≥2.5X upper limit in the absence of hepatic neoplastic disease.
- Laboratory: ANC <1,500/mm3, Hemoglobin < 8g/dL, platelet < 100,000/mm3, creatinine >2.0 mg/dL, urine uric acid > 1,000mg/d, urine oxalate > 60 mg/d, creatinine ≥2.0 mg/dL,
- Indiosyncratic intolerance of intravenous ascorbic acid such as acute hypersensitivity will be recorded.
- Unexpected or adverse events will be reported to the IRB and the FDA.

5.7 Part 1 Registration Guidelines

- All participants will be registered with Jefferson KCC and the Brind Center.
- All flow sheets and on and off study forms will be submitted to the Study Coordinator and Principal Investigator
- At the time of registration, the study subjects must have completed the Registration Form.
- Participants will not be registered if the IRB approval is not provided or is greater than 1 year prior to the date of registration. Participants must have informed consent.

5.8 Data Recording and Submission Schedule

- Data must be recorded according to the protocol requirements for all participants registered, whether or not assigned treatment is administered, including all participants deemed ineligible. Master forms are prepared by the Study Coordinator.
- Within 14 days of registration Study Coordinator must submit copies to subject charts of the following:
  - Registration Form
  - Pre-study Form
  - Study Specific Flow Sheet documenting history and physical, pre-study tests/exam results, first 7 days of protocol treatment and toxicity notations.
- After each infusion visit: Submit copies of the flow sheets and documentation of submission of samples.
- Within 14 days of completion or request to be removed from study:
- Submit copies of the Off Treatment Notice and Flow Sheet documenting the date/reason off treatment and summarizing inclusive dates of treatment and patient status.
- After off-treatment: Submit the Follow-up Form.

5.9 Additional Treatment Cycles

Subjects with metastatic adenocarcinoma of the pancreas will be enrolled in this phase I open-label clinical trial, receiving gemcitabine and vitamin C. In addition to ongoing safety assessments, response to therapy will be assessed after 8 weeks of therapy. Tumor response will be evaluated using Response Evaluation Criteria in Solid Tumors (RECIST) with modifications. Following this initial response assessment, subjects who meet the following conditions will be treated with additional cycles:

- Subjects with complete response (CR), partial response (PR) or stable disease (SD) will continue to receive the combination treatment until the first occurrence of either : 1) achievement of a confirmed CR; 2) clinical deterioration suggesting that no further benefit from treatment is likely; 3) progressive disease (PD) that is confirmed and then worsens: 4) development of a ≥ Grade 3 study drug-related adverse event (common terminology Criteria for Adverse Events {CTCAE}); 5) other intolerability of therapy; or 6) receipt of the maximum number of cycles.

- Subjects with PD that has been confirmed but is not worsening and with otherwise stable or improved clinical status should continue to be treated with study drug until there is further progression of clinical deterioration. ?

5.10 Intravenous Ascorbic Acid Procedure

The proposed protocol is similar to our current IRB-approved (IRB# 07U.21)/ FDA-approved (IND 77,486) protocol, and also consistent with that of the University of Kansas, the only other U.S. academic institution known to be conducting IV vitamin C research (IND# 65,805). The protocol also is within the dosage range of the phase I and II clinical trials recently published at McGill University in a mixed group of end stage cancer patients (Hoffer, 2006).

5.10.1 Doses

Doses will be reduced and/or delayed for toxicities. Toxicities will be graded using the NCI Common Terminology Criteria for Adverse Events Version 3.0. The toxic effects that may limit dose are: development of symptoms related to fluid overload (edema, dyspnea, orthopnea), renal insufficiency, acute tumor hemorrhage and necrosis. Dose adjustments are to be performed at the beginning of each week. For any grade 3 or 4 toxicity, the dose will be reduced to the next lowest dose level. If no recovery occurs after two weeks, the patient will go off protocol treatment. If two dose reductions are required, the patient will go off protocol treatment.

5.11 Concomitant Therapy

Throughout the study, investigators may prescribe concomitant medications or treatments deemed necessary to provide adequate supportive care except for those listed in the next section.

5.11.1 Proscribed Therapy During Study Period

The following therapies and treatment should not be administered while on study protocol:

- any investigational agent or other anti-tumor treatment (both standard and investigational) outside of protocol described therapy
- Herbal medications
- Elective major surgeries during the study through 30 days after the last dose of the protocol specified treatment.

5.12 General Study Procedures

Source documents, including but not limited to: radiological imaging including baseline scans must be stored and available for subsequent review. Unless otherwise specified all assessments will be done within 3 days of the planned visit. Signed and dated IRB informed consent must be obtained before any specific screening procedures are performed. Procedures that are part of routine care are not considered study specific procedures.

5.12.1 Screening Assessments

The following screening assessments must be performed and results available prior to enrollment:

- Review of inclusion and exclusion criteria
- Recording of concomitant medication and adverse events
- Medical history review, documentation of diagnosis and previous treatments including details of tumor diagnosis (eg, date of diagnosis, histology, stage at diagnosis and current stage) and most recent disease assessment
- Vital signs: resting pulse, resting respiration rate and temperature measurements, resting blood pressure (after the subject has been seated for at least 5 minutes)
- ECOG performance status
- Physical exam including weight and height
- Laboratory tests : Urinalysis, Hematology panels, G6PD status, Comprehensive chemistry panels including creatine kinase, magnesium, amylase, lipase, and lactate deyhdrogenase, fasting blood glucose, serum or urine pregnancy test for subjects of childbearing potential, PTT, INR
- ECG
- Radiological imaging to assess disease extent. Radiological assessments must include computerized tomography (CT) or magnetic resonance imaging (MRI) of the chest, abdomen, and pelvis and the modality selected should be the same throughout the study.

The subject should plan to receive first dose of gemcitabine and first dose of Vitamin C within 10 days after enrollment.

6.0 STUDY PARAMETERS

6.1 Cessation of Protocol Considerations

- Criteria for removal from protocol treatment: Progression of disease equals recurrent mass, new metastatic disease by examination or imaging (see section 6.2 below).

- For individuals, unacceptable toxicity (NCI Manual of Toxicities version 3.0) related to study drug (see section 5.4 above)

- Advance to ECOG Performance Status 3 or 4

- The study subject may withdraw from the study at any time for any reason.

- Study subjects who are found to be using more than 1 ppd of tobacco products will be removed from the protocol.

- Completion of treatment

- Termination by death will be documented in Notice-of-Death form

- Protocol will be terminated at any point if 5 subjects experience grade 4 toxicities as defined by the NCI Manual of Toxicities version 3.0 related to the ascorbic acid study drug

- All reasons for discontinuation of treatment will be documented in the flow sheets

- If more than 5 participants enrolled into the treatment arm experience Grade 4 toxicities by CTC criteria, further enrollment will be stopped.

6.2 Tumor Response Evaluation for continued cycles

6.2.1 Measurability of lesions

- Measurable disease: CT, MRI or other conventional technique with longest diameter 2 cm or greater in the axial plane or spiral CT with longest diameter 1 cm or greater. Ultrasound is unsuitable. It is noted that serial CT measurements can be difficult to compare, but care will be given to thin slice sections through the areas of identified measurable disease from scan to scan.
- Non-measurable disease: All other lesions including lesions too small to be considered measurable, pleural effusions, ascites, lymphangitis, pulmonitis, abdominal masses not confirmed and followed by imaging techniques, cystic lesions or disease documented by indirect evidence only (e.g., by lab values).

- Objective status at each evaluation: Objective Status is to be recorded at each evaluation. All measurable lesions up to a maximum of 10 lesions representative of all involved organs should be identified as target lesions at baseline. If there are more than 10 measurable lesions, the remaining are identified as non-target lesions and are included as non-measurable disease. Measurements must be provided for target measurable lesions, while presence or absence must be noted for non-target measurable and non-measurable disease.
- Complete Response (CR): Complete disappearance of all measurable and non-measurable disease. No new lesions. No disease related symptoms. Normalization of markers and other abnormal lab values. All disease must be assessed using the same technique as baseline.
- Partial Response (PR): Applies only to patients with at least one measurable lesion. Greater than or equal to 30% decrease under baseline of the sum of longest diameters of all target measurable lesions. No unequivocal progression of non-measurable disease. No new lesions. All target measurable lesions must be assessed using the same techniques as baseline.
- Stable: Does not qualify for CR, PR, Progression or Symptomatic Deterioration. All target measurable lesions must be assessed using the same techniques as baseline.
- Progression: One or more of the following must occur: 20% increase in the sum of longest diameters of target measurable lesions over smallest sum observed (over baseline if no decrease during therapy) using the same techniques as baseline. Unequivocal progression of non-measurable disease in the opinion of the treating physician (an explanation must be provided). Appearance of any new lesion/site. Death due to disease without prior documentation of progression and without symptomatic deterioration.
- Symptomatic deterioration: Global deterioration of health status requiring discontinuation of treatment without objective evidence of progression. Efforts should be made to obtain objective evidence of progression after discontinuation.
- Assessment inadequate, objective status unknown: Progression or symptomatic deterioration has not been documented, and one or more target measurable lesions have not been assessed or inconsistent assessment methods were used.

6.2.2 Objective status notes:

- Appearance or worsening of pleural effusions does not constitute unequivocal progression unless cytologically proven of neoplastic origin.
- If CR determination depends on a lesion for which the status is unclear by the required tests, it is recommended the residual lesion be investigated with biopsy or fine needle aspirate.

6.2.3 Performance Status:

- Patients will be graded according to the ECOG performance status scale (see Inclusion / Exclusion Criteria).

6.2.4 Time to Progression:

- From date of registration to date of first observation of progressive disease or death due to any cause.

7.0 STATISTICAL CONSIDERATIONS

We will initially enter 3 subjects at each dose. If none of the three experiences a dose-limiting toxicity we will proceed to the next dose. If one of the three experiences that level of toxicity, we will accrue 3 more subjects at that dose. If at any time there are two or more dose-limiting toxicities (in the 3-6 subjects) on a given dose, we will terminate accrual to the Phase I portion of that trial. No patient will be treated at a higher dose until the 3 or 6 patients have completed their toxicity evaluation period at the current dose. With this plan, a dose with a 50% or greater probability of causing a dose-limiting toxicity has at most a 12.5% chance of satisfying the conditions for dose escalation after the first 3 subjects and at least a 50% chance of stopping at 3. With the two-stages (3-6) together, there is at most a 17.2% chance of escalation.

While waiting for the 3 or 6 subjects accrued according to plan to complete their toxicity evaluation period, additional subjects may be accrued at the current dose. These additional subjects will not count towards the formal plan of stopping at two or more toxicity occurrences, but will contribute to the judgment as to the MTD.

Data analysis of phase I studies is descriptive. All estimates of dose-specific rates (e.g., response and toxicity) will be presented with corresponding confidence intervals using the exact method.

Analysis plan

Demographic information, including age and race will be tabulated. Descriptive statistics, including mean, median, standard deviations and ranges for all continuous measures will be tabulated and reported. Percentages and frequencies for all categorical measures will also be presented. Adverse events will be tabulated and listed according to severity. Two-sided 95% confidence intervals for rate of adverse events will be reported, based on exact methods, if number of events is small. Treatment efficacy will be estimated by progression free survival estimates (PFS). PFS will be reported according to Kaplan-Meier estimators, with 95% confidence intervals for median survival.

Secondary analysis of PFS will include Cox models of potential prognostic factors, using 2 – sided tests. Hazard ratios, 95% confidence intervals and p-values will be reported from these models.

7.1 Statistical Reference

Kindler HL, Aklilu M, Nattam S, Vokes EE. Arsenic Trioxide in patients with adneocarcinoma of the pancreas refractory to gemcitibine: a phase II trial of the University of Chicago Consortium. Amer J Clin Oncol 2008;31:553-6.

8.0 REFERENCES

Burris H, Storniolo AM. Assessing clinical benefit in the treatment of pancreas cancer: gemcitabine compared to 5-fluorouracil. Eur J Cancer 1997;33S:S18-22.

Chen Q, Espey MG, Krishna MC, et al. Pharmacologic ascorbic acid concentrations

selectively kill cancer cells: action as a pro-drug to deliver hydrogen peroxide to tissues. *Proc Natl Acad Sci U S A* 2005;102:13604-9.

Chen, Q, et al. Ascorbate in pharmacologic concentrations selectively generates ascorbate radical and hydrogen peroxide in extracellular fluid in vivo. *PNAS* 2007; 104: 8749-8754.

Chen Q, Espey MG, Sun AY, et al. Pharmacologic doses of ascorbate act as a prooxidant and decrease growth of aggressive tumor xenografts in mice. *PNAS* 2008;105:11105-11109.

Creagan ET, Moertel CG, O'Fallon JR, et al. Failure of high-dose vitamin C (ascorbic acid) therapy to benefit patient with advanced cancer. A controlled trial. *New Engl J Med* 1979; 301:687-90.

Drisko J., University of Kansas, Kansas City, Kansas. Phase I- II trial of gemcytabine with and without intravenous ascorbate for treatment of metastatic ovarian cancer; unpublished personal communication.

Drisko JA, Chapman J, Hunter VJ. The use of antioxidants with first-line chemotherapy in two cases of ovarian cancer. *J Amer Coll Nutr* 2003;22:118-23.

Heaney ML, Gardner JR, Karasavvas N, et al. Vitamin C antagonizes the cytotoxic effects of antineoplastic drugs. *Cancer Res* 2008;68:8031-8.

Hoffer LJ, Levine M, Assouline S, et al. Phase I clinical trial of i.v. ascorbic acid in advanced malignancy. *Ann Oncol* 2008; Epub ahead of print.

Hoffer, L.J., S. Assouline, D. Melnychuk, H. Sun, SJ Padayatty, M. Levine, W.H. Miller, Jr.. Lady Davis Institute for Medical Research, Jewish General Hospital, Montreal; Department of Oncology, Jewish General Hospital, Montreal; Center for Drug Evaluation and Research, US Food and Drug Agency, Rockville, MD; Molecular and Clinical Nutrition Section, US National Institutes of Health, Bethesda, MD
Phase I - II Trial of Intravenous Ascorbic Acid in Patients with Incurable Malignancies, 2006.

Jackson JA, Riordan HD, Hunninghake RE, Riordan NH. High-dose intravenous vitamin C and long-time survival of a patient with cancer of the head of the pancreas. *J.Orthomolecular Med.* 1995;10:87-8.

Jemal A, Siegel R, Ward E, et al. Cancer statistics, 2006. *CA Cancer J Clin* 2006;56:106-30.

Leung PY, Myashita K, young M, Tsao CS. Cytotoxic effect of ascorbate and its derivatives on cultured malignant and nonmalignant cell lines. *Anticancer Res* 1993; 13:475-80).

Levine, Mark et al. Vitamin C Pharmacokinetics in Healthy Volunteers: evidence for a Recommended Dietary Allowance. *Proc Natl Acad Sci USA 1996;*93:3704-9.

McKenna S, Eatock M. The medical management of pancreatic cancer: a review.

Oncologist; 2003; 8:149-60.

Moertal CG, FlemingTR, Creagan ET, et al. High-dose vitamin C versus placebo in the treatment of patients with advanced cancer who have had no prior chemotherapy. A randomized double-0blind comparison. *N Engl J Med* 1985; 312:137-41.

Moore MJ. Brief communication: a new combination in the treatment of advanced pancreatic cancer. *Semin Oncol* 2005; 32:5S-6S.

NCCN Clinical Practice Guidelines in Oncology. Pancreatic adenocarcinoma, 2008. National Comprehensive Cancer Network. 2008;www.nccn.org.

Padayatty SJ, Sun H, Wang Y, et al. Vitamin C pharmacokinetics: implications for oral and intravenous use. *Ann Intern Med* 2004; 140:533-7.

Padayatty SJ, Rjordan HD, Hewitt SM, et al. Intravenously administered vitamin C as cancer therapy: three cases. *CMAJ* 2006; 174(7):937-42.

Riordan HD, Jackson JA, Schultz M. Case study: high-dose intravenous vitamin C in the treatment of a patient with adenocarcinoma of the kidney. *J.Orthomolecular Med.* 1990;5:5-7.

Riordan NH, Riordan HD, Meng X, Li Y, Jackson JA. Intravenous ascorbate as a tumor cytotoxic chemotherapeutic agent. *Medical Hypotheses* 1995;44:207-13.

Riordan NH, Jackson JA, Riordan HD. Intravenous vitamin C in a terminal cancer patient. *J.Orthomolecular Med.* 1996;11:80-2.

Riordan HD, Riordan NH, Jackson JA, et al. Intravenous vitamin C as a chemotherapeutic agent: a report on clinical cases. *P R Health Sci* J 2004;23:115-8.

Sakagami, H, Satoh K, Hakeda Y, Kumegawa M. Apoptosis-inducing activity of vitamin C and vitamin K. *Cell Mol Biol* 2000; 46:129-43.

Wong K, Thompson C, Bailey RR, et al. Acute oxalate nephropathy after a massive intravenous dose of vitamin C. *Aust N Z J Med* 1994;24:410-1.

Yeo, CJ, Cameron JL, Sohn TA, et al. Six hundred fifty consecutive pancreaticoduodenectomies in the 1990s: pathology, complications, and outcomes. *Ann Surg* 1997;226:248-57.
